# Effect of shell as natural testosterone boosters in Sprague Dawley rats

**DOI:** 10.14202/vetworld.2019.1677-1681

**Published:** 2019-10-30

**Authors:** Pudji Astuti, Claude Mona Airin, Sarmin Sarmin, Alfarisa Nururrozi, Sri Harimurti

**Affiliations:** 1 Department of Physiology, Faculty of Veterinary Medicine, Universitas Gadjah Mada, Yogyakarta, Indonesia; 2 Department of Internal Medicine, Faculty of Veterinary Medicine, Universitas Gadjah Mada, Yogyakarta, Indonesia; 3 Department of Poultry Science, Faculty of Animal Science, Universitas Gadjah Mada, Yogyakarta, Indonesia.

**Keywords:** Cyp19, shell, testosterone, zinc

## Abstract

**Aim:**

This study aimed to evaluate the effect of shell supplementation on the regulation of male reproduction in rats

**Materials and Methods:**

The zinc (Zn) level of shell from blood clam (*Anadara granosa*), green mussel (*Perna viridis*), and conch shell (*Telescopium telescopium*) was analyzed. The highest Zn content shell was fed to male Sprague Dawley rats for 0, 9, 30, and 50 days at the dose of either 0.09 mg/200 g BW or 0.18 mg/200 g BW. To determine the testosterone levels, blood was collected through the infraorbitalis sinus just before the rat was sacrificed. Testicular and brain were also collected for Cyp19 aromatase receptor analysis.

**Results:**

The Zn level in the shell of blood clam, green mussel, and conch shell 61.55 mg/kg, 2.78 mg/kg, and 3.93 mg/kg, respectively. The testosterone level of T1 group receiving 0.18 mg/200 g BW for 0, 9, 30, and 50 days was 1.42±0.59, 2.15±1.58, 2.98±2.53, and 8.11±2.03 ng/mL, respectively. The testosterone level of T2 group receiving 0.09 mg/200 g BW for 0, 9, 30, and 50 days was 2.50±0.32, 1.25±0.60, 3.87±3.27, and 3.54±0.23 ng/mL, respectively. The T3 group receiving Na-CMC showed the level of testosterone at days 0, 9, 30, and 50 days was 0.77±0.22, 1.99±1.65, 4.12±0.07, and 2.19±1.30 ng/mL, respectively. Finally, the T4 group receiving Zn showed testosterone levels at days 0, 9, 30, and 50 days was 0.51±0.58, 2.24±3.16, 4.58±1.97, and 2.89±0.20 ng/mL, respectively. There was a significant difference (p<0.05) between the T1 group compared to the other groups. However, the absence of expression of Cyp19 aromatase both in Leydig cells and the brain indicated no conversion of testosterone to estradiol. To add, this finding showed the potential use of the shell to boost the testosterone level in male rats.

**Conclusion:**

Shell acted as an aromatase blocker to boost the testosterone level in male rats. This also indicates its promising application in birds to manipulate the quality of song and feather.

## Introduction

Shellfish is an abundant product, especially in Eastern Indonesia. In addition to its delicious taste, shellfish also contains numerous vitamins and nutrients. Approximate composition of fatty acid, amino acid profiles, and mineral content has been determined in oysters (*Crassostrea madrasensis*). The moisture protein, fat, carbohydrate, and ash content in oysters are 82.64%, 9.41%, 3.25%, 3.2%, and 1.01%, respectively. They are rich in macrominerals and trace elements, especially zinc (Zn) and selenium. Polyunsaturated fatty acids are the most common lipid including eicosapentaenoic acid, docosahexaenoic acid, and linoleic acid [1]. Zn is also very important and can be used as an aphrodisiac; a stimulant of enzymes, hormones, and the immune system. Since shellfish is abundant, the value of discarded shells waste has not much been explored.

If shellfish contains various nutrients, it is estimated that the shell may also contain the same nutrients, especially Zn. Zn is one of the macrominerals that function to increase testosterone levels both in humans and in mammals. It is indicated that Zn works by reducing the aromatase enzyme Cyp19, an enzyme that converts testosterone to estradiol. Chu *et al*. [2] study on Leydig cells in mice found that Zn transporters (ZnT7) may play an important role in the regulation of testosterone synthesis by modulating steroidogenic (STARs) enzymes and may represent as therapeutic target in testosterone deficiency. In singing birds, testosterone levels influence the quality of sound and feathers. Sankako *et al*. [3] reported that testosterone level will increase if aromatase enzyme is blocked since the conversion of testosterone to estrogen is inhibited. Salehi *et al*. [4] found that testosterone level of certain snakes living in four seasons is greatly affected by spermatogenesis and body weight. Reed *et* *al*. [5] found that Zn influenced the digestive system of chickens. Birds with Zn deficiency tend to experience digestive disorders.

This study aimed to determine the effect of shellfish supplementation on the regulation of male rat reproduction by measuring the level of testosterone in serum, Cyp19 expression in testicular and brain by immunohistochemistry. If the results showing a significant effect, it will then apply to sing birds to improve the quality of sound and feathers.

## Materials and Methods

### Ethical approval

The Ethics Committee of the Integrated Testing and Research Laboratory, Universitas Gadjah Mada (UGM), certificate number 00023/04/LPPT/IV/2018 approved all procedures.

### Shell origin and analysis

Three shells from blood clams (*Anadara granosa*), green mussels (*Perna viridis*), and conch shells (*Telescopium telescopium*) were analyzed. The shells were collected from Samas and Glagah Beach, Yogyakarta Special Region, Indonesia. Identification of shellfish species was carried out at the Department of Biology, Universitas Gadjah Mada (UGM), Yogyakarta, Indonesia. The production of shell powder and the analysis of macrominerals contents were carried out using inductively coupled plasma at the Integrated Testing and Research Laboratory, UGM, Yogyakarta, Indonesia. Shell with the highest Zn content then continued for *in vivo* analysis.

### Animals for *in vivo* study

The animals used in the study were Sprague Dawley male rats at 1 month of age. Animals were divided into four groups: T1 received 0.18 mg/200 g BW shell powder of *A. granosa*; T2 received 0.09 mg/200 g BW of *A. granosa*; T3 received Na-CMC; and T4 received Zn 0.09/200 g BW orally. The blood was withdrawn by infraorbital vein at 9, 30, and 50 days post-treatment; then, animals were sacrificed.

### Testosterone analysis

The serum was separated through centrifugation at 3000 rpm for 15 min and stored at −20°C before used. Samples were then analyzed using ELISA method.

### Immunohistochemical staining methods

Collected testicular and brain were fixed in 10% PBS-formalin solution for 18-24 h then embedded in paraffin. The paraffin blocks were then cut to a thickness of 4 μ, placed on slides, coated with poly-L-Lysine (Sigma^®^), and probed with monoclonal antibody Cyp19 aromatase (C-16) SC-14245 (Santa Cruz Biotechnology) at 1:100 of dilution. Subsequently, secondary biotinylated antibody at 1:100 dilution was applied, labeled with strept-enzyme-avidin peroxidase, and mounted with Canada balsam. Brown stained cells indicated the expression of Cyp19 aromatase.

### Statistical analysis

Data were analyzed using SPSS statistical software (IBM, USA) and average data were compared using one-way ANOVA and Duncan test with a confidence level of 0.05. The immunohistochemical staining results of Cyp19 receptors from testicular and brain were analyzed descriptively.

## Results

### Macromineral contents of shell

The macrominerals contain in shell and meat of shellfish used in this research are listed in Table-1.

**Table 1:** The macrominerals contain in shell and meat of blood clam, green mussel, and conch shell.

Oyster shell	Zn (mg/kg)	Mg (mg/kg)	Ca (mg/dL)	Na (mg/kg)	Fe (mg/kg)	K (mg/kg)
*A. granosa*	61.55	1666.09	41.4	9262.98	600.54	369.29
*P. viridis*	2.78	141.37	55.58	8385.29	3.99	174.23
*T. telescopium*	3.93	151.23	47.15	7793.41	8.5	164.91

**Oyster meat**	**Zn (mg/kg)**	**Mg (mg/kg)**	**Ca (mg/dL)**	**Na (mg/kg)**	**Fe (mg/kg)**	**K (mg/kg)**

*A. granosa*	102.27	14.08	370.81	618.77	57.78	334.41
*P. viridis*	64.23	14.06	1297.58	463.76	771.34	281.14
*T. telescopium*	169.68	164.49	615.18	1558.05	32.05	1394.75

*A. granosa*=*Anadara granosa*, *P. viridis*=*Perna viridis*, *T. telescopium*=*Telescopium telescopium*, Zn=Zinc

Although clams contained various types of macromineral, only Zn will be further investigated as a potential aromatase blocker. However, Zn analysis showed *Anadara granose* shell contained the highest Zn compared to others.

### The effect of shell to testosterone level

Zn contained oyster meat can improve testosterone levels in humans [6]. It is known that aromatization plays an important role in the testosterone signaling pathway of the brain. Furthermore, estrogen metabolite along with testosterone regulates and activates masculine neural circuit [7].

The result showed post-administration of shell powder for 50 days, the testosterone level was increased either in the control or treatment group (Table-2). Significant difference was observed between control (Na-CMC) and T1 (Zn 0.18 mg/200 g) and T4 (0.09 mg/200 g) group.

**Table 2:** The average of testosterone levels at days 0, 9, 30, and 50 after treatment.

Treatment groups	Average±SE level of testosterone on days (ng/mL)			

	0	9	30	50
T1 (Zn 0.18 mg/200 g)	1.42±0.59	2.15±1.58	2.98±2.53	8.11±2.03*
T2 (Zn 0.09 mg/200 g)	2.50±0.32	1.25±0.60	3.87±3.27	3.54±0.23
T3 (Na-CMC)	0.77±0.22	1.99±1.65	4.12±0.07	2.19±1.30
T4 (Zn 0.09 mg/200 g)	0.51±0.58	2.24±3.16	4.58±1.97	2.89±0.20*

* Significantly different (*P*>0.05). Zn=Zinc

### The expression of Cyp19 aromatase in Leydig cells

**Figure-1:**
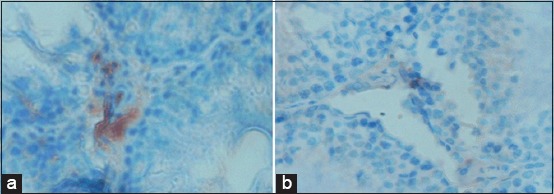
Expression of Cyp19 aromatase in Leydig cells of the control group (a). Negative expression of Cyp19 aromatase in T1 receiving 0.18 mg/200 g BW (b). Aromatase expression indicated inhibition of testosterone conversion activity.

The Cyp19 aromatase of Leydig cells in the control group (Na-CMC) was highly expressed (Figure-1a). However, Cyp19 aromatase in the treatment group at 0.18 mg/200 g was poorly expressed (Figure-1b). Moreover, a similar result also obtained from the pure Zn-treated group.

According to Sankako *et al*. [3], Zn works as an aromatase blocker; therefore, the conversion of testosterone into estrogen is inhibited. This was confirmed in the level of testosterone between treatments compared to control after 50 days of shell administration, which was significantly different (p<0.05).

### The expression of Cyp19 aromatase in brain

**Figure-2:**
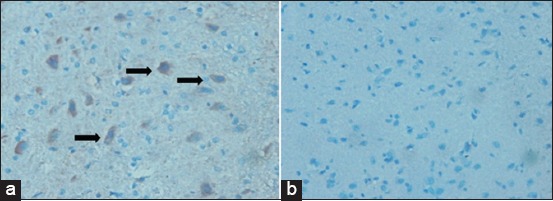
Negative expression of Cyp19 aromatase in the brain of the control group (a), a group of shellfish with a dose of 0.18 mg/BW (b), where no expression of Cyp19 aromatase indicated no conversion of testosterone to estrogen.

Aromatase can also be found in the vertebrate brain from fish to primate, particularly in the brain cell related to hypothalamus reproduction. This research showed a high expression of Cyp19 aromatase receptors in the brain cells of the control group, vice versa of the treatment group (Figure-2). Negative expression of Cyp19 aromatase indicated the role of the shell as aromatase blocker resulted in a high level of testosterone.

### The effects of Cyp19 aromatase on spermatogenesis

**Figure-3:**
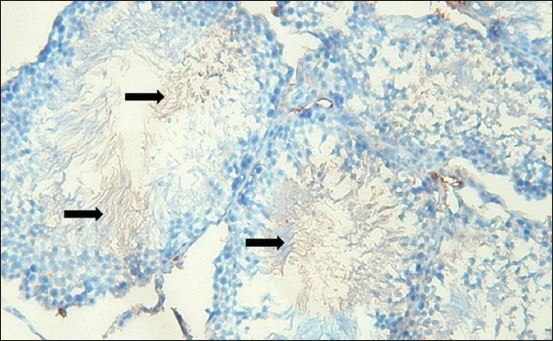
The expression of aromatase Cyp19 on spermatozoa (brown color) indicating that estradiol is needed in the survival process of spermatozoa.

The Cyp19 aromatase in spermatozoa cell after administration of shell was persistently expressed, although poor expression was showed in Leydig and brain (Figure-3). This indicated the use of estradiol in the development of the reproduction system.

Ninety years ago, Hess and Cooke [8] reported that estradiol for men is considered useless, however, now has been proven that the mainestrogen receptor 1 (ESR1) plays an important role in male fertility and the development of efferent channels, epididymis, prostate, and spermatozoa maturation [9]. Conversely, women also respond to exogenous testosterone, i.e., growth of body hair even though short [10].

## Discussion

Zn supplement plays an important role as an antioxidant by protecting the concentration of sulfhydryl (SH). Zn-deficient mice have lower testosterone levels compared to normal mice [11]. Furthermore, a low level of testosterone is associated with infertility in humans [12]. Besides the reduction of testosterone level, Zn deficiency is associated with impaired spermatogenesis due to the elevation of oxidative stressor and apoptosis. Zn is capable of increasing testosterone through aromatase inhibition mechanism by blocking the conversion of testosterone to estradiol [3]. Letrozole, an aromatase blocker, can increase testosterone both in rats and old rooster in different pathways. In rats, letrozole can increase of gonadotropins due to lack of estrogen [13,14]. Similar results to Taxadrol (aromatase blocker), it used to be traditional hormone therapy to increase testosterone [15]. This finding shows that Zn plays an important role in male reproduction process [16]. The induction of testosterone to a female robin bird is correlated to the cellular differentiation process in the higher vocal center [17].

### The effect of oyster shells on expression Cyp19 aromatase in Leydig cells

Aromatase is known as estrogen synthetase, a key enzyme in estrogen biosynthesis. The enzyme is located within the endoplasmic reticulum encoded by the Cyp19A gene [18]. This gene belongs to the family of the CYP gene, which encodes a class of active enzymes in the hydroxylation of endogenous and exogenous substances [19]. Aromatase receptors are located in bone, brain, and adipose tissues [9]. The administration of exogenous testosterone or letrozole aromatase inhibitor can improve spermatogenesis disorder which affecting testicular phenotype [20]. Adeldust *et al*. [21] stated that animals treated with letrozole showed a higher number of spermatozoa cells in the tubules and seminiferous epididymis compared to control animals. Paracrine signaling of the aromatase enzyme is very important in spermatogenesis [9].

Testosterone is naturally produced in Leydig cells. However, this research showed weak expression of Cyp19 aromatase in the control group, which indicated low estrogen but high testosterone level (Figure-3). Boulanger *et al*. [20] reported a reduction of testosterone and increment of estradiol levels up to 3 times in Celf1-/- mouse testicles during hyperactive aromatase activity. Rosati *et al*. [22] reported similar conditions of aromatase P450 presentation in somatic cells and testicular germinal *Podarcis sicula* (spermatids and spermatozoa) except in early autumn where aromatase is only evident in Leydig cell. Ogunlesi *et al*. [23] reported *Momordica charantia*, *Neoregelia laevis*, and *Rauvolfia vomitoria* as sources of Zn and Vitamin C. These plants have been used to treat infertility in men.

### The effect of oyster shells on expression of Cyp19 aromatase in brain

Testosterone can regulate physical activity in male rats through the dopamine agonist pathway by both directly through the androgen receptor (AR) and indirectly through the aromatization of testosterone to estrogen [24]. Antonio-Cabrera and Paredes [25] reported the induction of mating in male rats implanted with testosterone or estradiol at the medial preoptic region. This indicated that a lack of sexual behavior is associated with hormonal changes. The highest aromatase activity is in the neuroendocrine region, which consists of the posteromedial amygdala nucleus, the encapsulation region of the terminal stria nucleus, the bed nucleus of the stria terminalis (BNST), the ventrolateral portion of the ventromedial hypothalamic nucleus, and the central component of the medial preoptic nucleus. Besides, endocrine circuits in the brain also contain estrogen-ARs [26].

### The effect of Cyp19 aromatase on spermatogenesis

Kumar and Singh [27] proposed the aromatase blocker effect of Zn in tackling male infertility. Furthermore, Zn deficiency inhibits spermatogenesis and increases abnormal sperms. It also proved to have a negative effect on the concentration of testosterone in serum. Thus, Zn is considered beneficial for prevention, treatment, and diagnostic marker in male infertility [28]. Stocco [9] reported high aromatase activity in mice Sertoli cells before sexual maturity then become prominent in Leydig cells of adult animals. Schulster *et al*. [29] reported a complex balance of testosterone, estradiol aromatase, and ER in testicular, penis, and brain. This confirms that estrogen is required and well regulated in men. ER and aromatase shared topographical locations with pheromones in the brain and it is clear that estrogen contributes to sexual early development and behavior. The absence of estradiol ESR1 and 17 beta membrane fractions can induce male reproduction abnormalities to infertility [7]. The testes produce large amounts of estradiol associated with aromatase in several cell types. Stocco [9] reported that aromatase mRNA can be detected in spermatogonia gonocytes, preleptotene spermatocytes, and at all stages of the development of cell germ [18]. Low testosterone levels can cause abnormalities of muscle and bone development, loss of strength, energy, and sexual drive together with sperm count declined [30]. Testosterone also affecting the reproductive system of female birds by increasing the secretion of the luteinizing hormone, follicle growth, laying eggs, and the need to build nests [31]. However, the age and environment are important factors that modulate the effects of both endogenous and exogenous sex hormones [32]. Hau *et al*. [33] stated that the peak of testosterone during the mating season is higher in short-lived species with high mating efforts because this hormone is known to promote male fecundity.

## Conclusion

Administration of shell powder at 0.18 mg/200 g of body weight can increase testosterone levels in mice and block the expression of Cyp19 aromatase. Shell powder can be employed to promote testosterone level through aromatase blocker mechanism. Since estradiol is required in the development of vas deferens, epididymis, and prostate gland, aromatase blocker administration is suggested only for adult animals.

## Authors’ Contributions

PA planned the study and drafted the manuscript, designed the experiment protocol. CMA and AN collected and analyzed samples. SS revised the manuscript under the supervision of PA and SH. All authors read and approved the final manuscript.
